# The effectiveness of reminiscence therapy on the symptom management, the life satisfaction, and the self-transcendence in palliative care patients: a randomized controlled trial

**DOI:** 10.1007/s00520-024-08626-9

**Published:** 2024-06-14

**Authors:** Canan Bozkurt, Yasemin Yildirim

**Affiliations:** 1 Nursing Department, Faculty of Health Sciences, Bandirma Onyedi Eylul University İhsaniye District, Kurtuluş Street, Nu:98, Bandirma, Balıkesir, 10200 Turkey; 2 Department of Internal Medicine Nursing, Faculty of Nursing, Ege University, Izmir, Turkey

**Keywords:** Reminiscence therapy, Palliative care, Symptom assessment, Life satisfaction, Self-transcendence

## Abstract

**Purpose:**

To examine the effect of individualized reminiscence therapy on the management of global distress and physical and psychological symptoms, life satisfaction and self-transcendence levels of palliative care patients.

**Methods:**

In a single-center palliative care service in western Turkey, 48 patients without cognitive impairment and able to communicate were included in the study. However, 44 patients completed the study. Patients who met the inclusion criteria were randomly assigned to the reminiscence therapy (intervention), unstructured social interviewing (placebo), and control groups (16 people for each group) before the start of the study. The sessions for the interview and placebo groups were conducted face-to-face in the patient's room (while the patient was sitting or lying down) for 15 days (2 weeks), every other day, for a total of eight sessions (each session was approximately 30 min). Data collection instruments—the Memorial Symptom Assessment Scale, the Contentment with Life Assessment Scale, and the Self-Transcendence Scale—were collected at baseline (first day) and after the intervention (day 15^th^). Statistical significance level was accepted as *p* < 0.05.

**Results:**

There was no decrease in physical and total symptom burden (*p* > 0.05). There were significant reductions in general distress and psychological symptoms in the intervention and placebo groups within the group (*p* < 0.05), but there were no significant differences between the control group and all groups when compared (*p* > 0.05). Group × time interactions were statistically significant for life satisfaction and self-transcendence (*p* < 0.001), and there was a substantial increase in the intervention group compared to the other groups.

**Conclusion:**

It may be recommended that reminiscence therapy intervention be included in routine nursing care as it may contribute positively to the psychological recovery of palliative care patients approaching the end of life.

**Trial registration:**

*ClinicalTrails.gov* (Registration number: NCT05242016). Prospectively registered on 1 February 2022.

## Introduction

Based on Erik Erikson’s theory of psychosocial development, the gerontologist and psychiatrist Butler, in his work “Life Review”, argued that it is possible to analyze the past by integrating and interpreting experiences [1]. After Butler, who first applied the word reminiscence to this context, studies have emerged of the practice of reminiscence therapy (RT) involving the structuring of the review process of life [2]. Although it initially appeared as a psychoanalytic concept, RT has been used as a nursing component in long-term care institutions. Nurses began using RT in the late 1960s and published their experiences in the 1970s. At that time, the aims of nurses in applying RT were to help older people to view their experiences from a different perspective and prepare for death [2–4]. In fact, this practice has been mostly used in older people and people with dementia [4, 5]. In recent years, it has been thought that it can improve mental health in people approaching the end of life and struggling with difficult diseases or cancer, and studies have gained weight in this direction [6–8]. However, although there are studies on RT applied to cancer patients, there is a limited number of studies covering all palliative care patients. In fact, in 2019, Kwan et al. conducted the first randomized controlled trial testing the first short-term life review intervention for individuals receiving palliative care [9]. The authors noted that this intervention could help palliative care patients approaching the end of their lives, regardless of their chronological age, to face the same developmental crises and decline in functioning as older adults [9]. In this context, RT, which is beneficial for reducing older adults’ concerns about finding meaning in life and spiritual concerns, may also benefit palliative care patients.

In the early 1990s, it was recognized that patients in the late stages of many chronic terminal diseases also benefit from palliative care, and the field was expanded accordingly. Palliative care is a philosophy of care in which all treatments are aimed at preventing/reducing pain and improving quality of life and are provided with a simultaneous multidisciplinary approach. This is achieved by early detection of all physical, psychosocial, and spiritual symptoms, especially pain, of individuals and their families who face problems arising from life-threatening diseases [10, 11]. In addition to symptom control, one of the goals of palliative care is supportive care. Supportive care is care that helps the patient and family cope with the illness and its treatment. It helps the patient maximize the benefits of their treatment and helps the patient—and therefore their family—feel as good as possible. It has equal priority with diagnosis and treatment [12]. In this context, complementary and supportive therapies to be applied in addition to pharmacological treatment can contribute to the positive development of the patient’s mental health, coping with the disease and self-actualization, especially in end-of-life care.

Self-actualization was defined by Maslow as the highest, most inclusive, or holistic consciousness that goes beyond the self and was added as a motivational step in the “Hierarchy of Needs” model [13]. Over time, many nursing theorists also mentioned the concept of self-actualization and finally a middle-class nursing theory was developed by Reed under the name of “Self-Transcendence Theory” [14]. According to Reed, self-transcendence is associated with maintaining well-being even when faced with loss or life-limiting situations. Self-transcendence is an internal capacity that enables individuals exposed to stressful life events to find a new purpose and meaning in life. Existing research on self-transcendence focuses on people with life-threatening or life-limiting illnesses [7, 14, 15]. In this context, Reed reported that in nursing care, meditation, prayer, visualization, self-reflection, and journaling, as well as life review and RT can be used as self-transcendence techniques that can facilitate nurses’ recognition of healing patterns in their patients and support individuals’ well-being potential [14, 15]. In the past, Ebersole emphasized that identification, socialization, intergenerational sharing, memory stimulation, and the concept of self-transcendence are therapeutic factors of RT [2–4].

The “life-threatening or life-limiting diseases” mentioned by Reed in his “self-transcendence theory” are the focus of palliative care [14]. It is thought that alleviating/eliminating the symptoms seen in the process that results in death in palliative care patients can increase the quality of life, and that the patient can reach life satisfaction and find the opportunity to overcome themselves by making sense of the past and transforming negative perspectives into positive ones. In this context, this study aims to examine the effect of RT on palliative care patients’ management of global distress and physical and psychological symptoms, life satisfaction, and self-transcendence.

## Methods

### Design

**Fig. 1:**
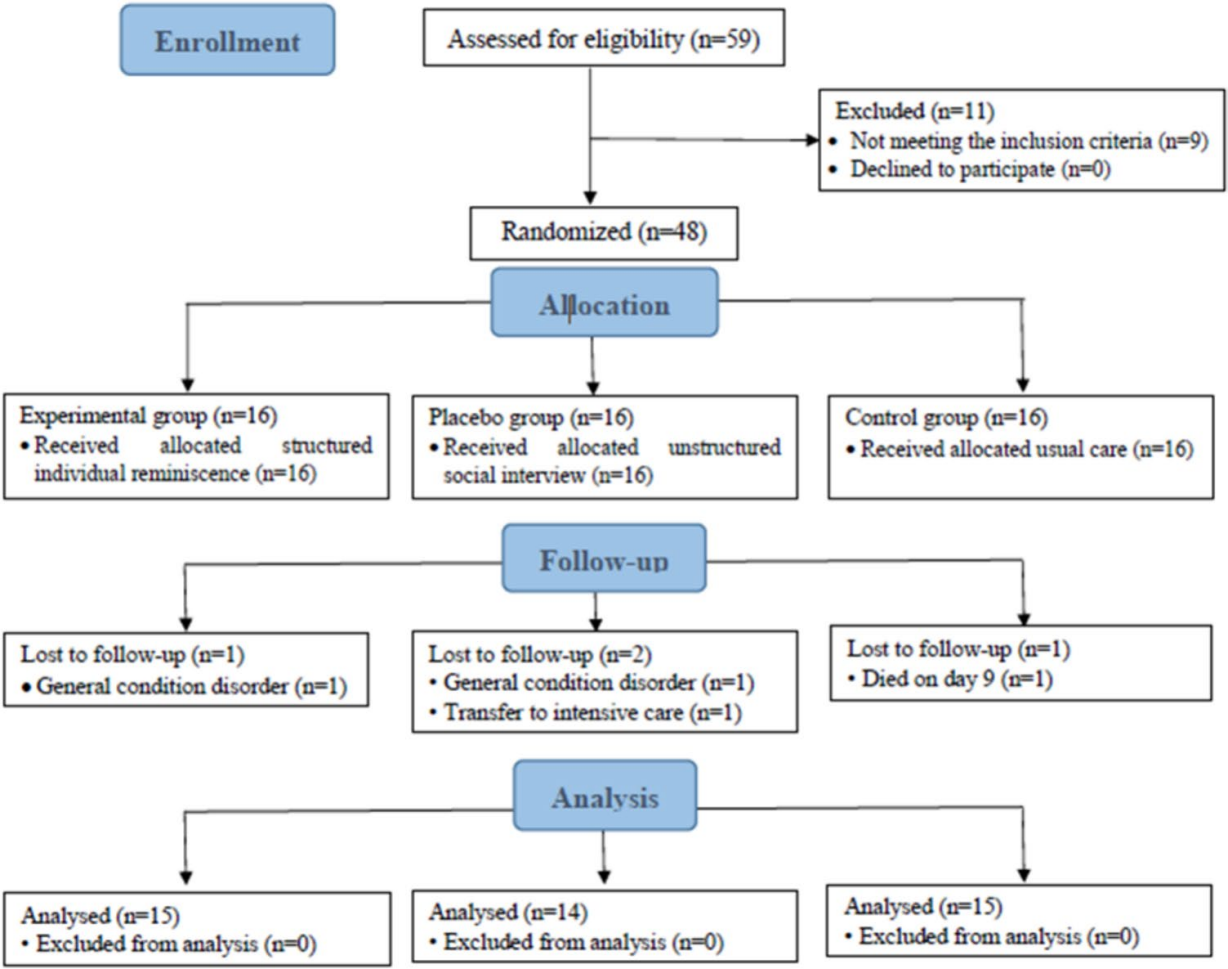
CONSORT flow chart

This study was designed as a randomized, three-arm placebo-controlled trial, unblinded, and conducted according to the Consolidated Standards for Reporting Trials (CONSORT) (Fig. 1).

### Setting and sample

This study was conducted on patients treated in the palliative care unit of a public hospital in Turkey between November 2021 and May 2022.

To determine the required sample size, the lowest number of cases to be included in the study was determined to be 33, with 95% confidence (1-α), 80% test power (1-β), and an effect size of *f* = 0.514 in the initial power analysis; the minimum required number of patients in a group was calculated as 11 [16]. Considering the possible losses, it was planned to include 16 patients in each group, and 48 patients who met the inclusion criteria at the time of the study were included. However, 44 patients completed the study.

In the post hoc power analysis performed after the study, the effect size was found to be *f* = 0.556 according to the self-transcendence scale, and the power of the study (1-β) was 98.6% with 95% confidence (1-α).

The inclusion criteria were (a) hospitalized in the palliative care unit, (b) no cognitive impairment, oriented, and able to communicate, (c) over 18 years old, (d) voluntary participation in the research, (e) able to speak and understand Turkish, and (f) no hearing or vision problem patients. The exclusion criteria were patients (a) with a clinical diagnosis of dementia, agitation, and delirium and (b) who are scheduled to be referred and/or discharged to another unit/institution within the estimated period of investigation (15 days) by the physician and do not have a life expectancy [9].

### Randomization and blinding

The stratified block randomization method was used to select the groups. The patients included in the three groups (RT group-intervention, unstructured social interviewing [UCI] group-placebo, and control group) were blocked after stratification by age and gender by the researchers. Stratified randomization was performed to eliminate the significant effects of age (< 65 years/ ≥ 65 years) and gender (female/male) factors on perceptions of reminiscence and to increase the reliability of the results. Patients were divided into four categories according to age and gender ([A] “female and < 65 years old”, [B] “female and ≥ 65 years old”, [C] “male and < 65 years old”), [D] “male and ≥ 65 years old”).

Random numbers generated by a web-based computer program (https://www.random.org) were used in the randomization protocol. All patients included in the study continued to be given standard nursing care during this time.

Researchers and participants were not blinded. To avoid selective reporting bias, data were performed and reported in the SPSS software package by an unbiased expert independent of the study. In this context, it can be said that the study was single-blind because the person who made the analysis results did not know the groups.

### Procedures

After obtaining written consent from the patients who met the inclusion criteria and agreed to participate in the study, research started. Reminiscence therapy was conducted by the researcher, a trained nurse with a master’s degree in internal medicine nursing. In the country where the study was conducted, there was no specific training on RT; however, a 72-h professional coaching training and certificate was obtained, which included RT. The sessions for the interview and placebo groups were conducted face-to-face in the patient’s room (while the patient was sitting or lying down) for 15 days (2 weeks), every other day, for a total of eight sessions (each session was approximately 30 min).

### Intervention group

For the patients included in the structured reminiscence (intervention) group, in the first session, general topics specific to the participant’s life and characteristics (such as living with a mother/father, education, marital status, number of children, and whether the patient had worked in any job or never worked) were determined. In each session, the patient’s relatives were asked to provide an album, photograph, or object that would facilitate remembering in each session. The second session was focused on childhood and family life; the third session school life; the fourth session entertainment, activities, and hobbies; the fifth session food and drink; the sixth session celebrations (such as birthdays, weddings, births, holidays, and special days); the seventh session when the patient was happiest and proudest; and the eighth and last session entailed a general evaluation made using a summary of the previous sessions, and their feelings about the sessions were shared. During this period, standard nursing care was continued.

### Placebo group

For the patients included in the placebo group, unstructured social interviews were conducted during each session, for which the topic for the next session was not predetermined but rather selected in response to the patient’s request, such as current issues not related to the past or information about the individual’s diseases or care, if any. During this period, standard nursing care was continued.

### Control group

The patients in the control group did not undergo any treatment related to the study, and standard nursing care was continued.

### Data collection

When all the patients were recruited for initial randomization (first day—baseline), a patient identification form, the Eastern Cooperative Oncology Group (ECOG) Performance Scale, the Memorial Symptom Assessment Scale (MSAS), the Contentment with Life Assessment Scale (CLAS), and the Self-Transcendence Scale (STS) were administered. After the intervention (day 15^th^), data collection forms consisting only of the MSAS, CLAS, and STS were administered and collected by the researcher nurse using a face-to-face interview technique. The scales are based on self-report. However, the data was collected by the researcher because the patients were palliative care patients with high performance scales, experienced many symptoms, were not healthy patients, and had difficulty reading and writing.

### Outcomes and measures

The patient identification form was developed by the researchers concerning the literature [9, 16]. This form includes questions about the sociodemographic (age, gender, marital status, etc.) and health/illness status (length of stay in the palliative care unit, the disease requiring hospitalization, and the year of illness) of the patients.

The Eastern Cooperative Oncology Group (ECOG) Performance Scale is a performance measurement tool published by Oken et al. in 1982 and used for performance evaluation in cancer patients. On this scale, the patient is given 0 points if their health is excellent and five points if they are dead [17]. The patient’s performance status is expressed with a score appropriate to the defined functional capacity. This performance score is based solely on the observation of the clinician or researcher.

The Memorial Symptom Assessment Scale (MSAS) evaluates the frequency, severity, and distress of common symptoms experienced in the previous week by individuals diagnosed with cancer [18]. A validity and reliability study of the Turkish version of the scale was carried out by Yıldırım et al. (2011). On the scale, there are 32 symptoms, which are evaluated in two parts [19]. The subdimensions of the scale include the Global Distress Index (MSAS-GDI), Physical Symptom Distress Score (MSAS-Physical), and Psychological Symptom Distress Score (MSAS-Psycho) [18, 19]. In the Turkish validity and reliability study, Cronbach’s alpha reliability coefficients were found to be 0.75, 0.75, and 0.71 for the MSAS-GDI, MSAS-Physical, and MSAS-Psycho subscales, respectively. The Cronbach’s alpha reliability coefficient for the total MSAS was found to be 0.84 [19]. In the present study, the Cronbach’s alpha reliability coefficient of the scale was found to be 0.74 for MSAS-GDI, 0.73 for MSAS-Physical, 0.78 for MSAS-Psycho, and 0.89 for total MSAS.

The Contentment with Life Assessment Scale (CLAS), developed by Lavallee et al. consists of five items rated on a 7-point Likert-type scale [20]. Akın and Yalnız conducted the Turkish validity and reliability studies of the scale in 2015 [21]. The lowest score that can be obtained from the scale is 7 and the highest score is 35, indicating that the higher the score, the higher the satisfaction. In the Turkish validity and reliability study, Cronbach’s alpha internal consistency reliability coefficient was reported as 0.73 [21]. In this study, Cronbach’s alpha coefficient was calculated as 0.77.

The Self-Transcendence Scale (STS), developed by Reed, consists of 15 items rated on a four-point Likert-type scale [14]. The original scale consisted of two subscales: interpersonal and internal mood, but the overall score of the scale gave better values in the Turkish version adapted by Sarıçam [22]. The score range to be obtained from the scale is between 15 and 60, and a high score indicates a high level of self-transcendence. In the Turkish validity and reliability study of the scale, Cronbach’s alpha internal consistency reliability coefficient was 0.87 [22]; in this study, Cronbach’s alpha coefficient was 0.82.

### Data analysis

The data obtained from the research were analyzed using the IBM SPSS Statistics 23 (SPSS Inc., Chicago, IL) program package with appropriate statistical methods on a computer belonging to the researcher. The data’s conformity to normality was checked with the Kolmogorov–Smirnov test; the assessment was made by considering the skewness/kurtosis values. Descriptive statistics were used for the participants’ demographic and health/illness characteristics. Mean, standard deviation, and lowest-maximum values were used for continuous variables; frequency and percentage were used for categorical variables.

Pearson’s chi-square test or Fisher’s exact chi-square test was used to examine the relationship between two categorical variables. In comparison tests, paired dependent sample *t* tests, one-way ANOVA for variables with normal distribution, the Wilcoxon signed-rank test, and the Kruskal–Wallis *H* test were used for non-normally distributed variables. The Bonferroni test was applied for multiple group comparisons (post hoc). In the two-way ANOVA analysis, evaluations were made according to the Mauchly sphericity test result when the sphericity assumption was met and according to the Greenhouse–Geisser result when the sphericity assumption was not met. A post hoc analysis was performed when the group*time interaction yielded a statistically significant output. The results were evaluated at 95% confidence and *p* < 0.05 significance levels.

### Ethical considerations

Ethics committee approval was obtained from the Ege University Medical Research Ethics Committee (21–7.1 T/29) for the study to be carried out; institutional permission was obtained from the Provincial Health Directorate were obtained in accordance with the Declaration of Helsinki. Permission from the authors was obtained to use the scales. The patients were informed about the study with the informed consent form. Those who agreed to participate and met the inclusion criteria were included in the study. No harmful drugs or interventions were applied to the patients. In addition, the study protocol was registered on *ClinicalTrails.gov* (Registration number: NCT05242016, prospectively registered on 01 February 2022).

## Results

Figure 1 shows the CONSORT flow chart. A total 59 participants were screened for eligibility, of which 48 met the inclusion criteria and were recruited. Finally, 48 participants (16 in each group), 44 of whom completed the study (four did not due to death [*n* = 1], transfer to intensive care unit [*n* = 1], and general condition [*n* = 2]), were recruited.

### Patients’ characteristics

The sociodemographic and disease-related characteristics of the palliative care patients included in the study were analyzed. The mean age is 64.93 (SD 3.69) and ranges from 53 to 71. The most common diseases causing patients to be admitted to the palliative care unit were cancer (70.5%). The performance level of 63.6% of the patients was “3” according to the ECOG scale. When these features were compared between the groups, there was no significant difference (*p* > 0.05) and showed a homogeneous distribution (Table 1).

**Table 1:** Patients’ demographic and clinical characteristics

Variables	Total *n* = 44	Intervention *n* = 15	Placebo *n* = 14	Control *n* = 15	*p* value
Age (years)					
Mean, SD	64.93, 3.69	65.67, 3.67	64.57, 4.15	65.60, 2.56	0.655^a^
%95 CI (LL, UL)	64.24, 66.35	63.63, 67.70	62.18, 66.97	64.18, 67.02	
Age groups, *n* (%)					0.844^b^
< 65 years	13 (29.5)	4 (26.7)	4 (28.6)	5 (33.3)	
≥ 65 years	31 (70.5)	11 (73.3)	10 (71.4)	10 (66.7)	
Gender, *n* (%)					
Female	17 (38.6)	5 (33.3)	6 (42.9)	6 (40.0)	0.853^b^
Male	27 (61.4)	10 (66.7)	8 (57.1)	9 (60.0)	
Marital status, *n* (%)					
Married	38 (86.4)	14 (93.3)	11 (78.6)	13 (86.7)	0.796^b^
Single/widowed/divorced	6 (13.6)	1 (6.7)	3 (21.4)	2 (13.3)	
Primer disease, *n* (%)					
Cancer	31 (70.5)	10 (66.7)	11 (78.6)	10 (66.7)	0.829^c^
Cardiovascular system	5 (11.4)	2 (13.3)	1 (7.1)	2 (13.3)	
Neurological system	3 (6.8)	2 (13.3)	1 (7.1)	–-	
Respiratory system	3 (6.8)	–-	1 (7.1)	2 (13.3)	
Urinary system	2 (4.5)	1 (6.7)	–-	1 (6.7)	
Cancer localization (*n* = 31), *n* (%)					
Genitourinary	8 (25.8)	2 (20)	3 (27.3)	3 (30.0)	0.817^b^
Respiratory	7 (22.6)	3 (30)	2 (18.2)	2 (20.0)	
Gastrointestinal	7 (22.6)	2 (20)	2 (18.2)	3 (30.0)	
Breast	5 (16.2)	2 (20)	2 (18.2)	1 (10.0)	
Hematological	4 (12.9)	1 (10)	2 (18.2)	1 (10.0)	
Duration of the disease (years)					
Mean, SD	5.14, 3.03	4.27, 1.62	5.71, 2.97	5.47, 3.19	0.465 ^a^
95% CI (LL, UL)	4.12, 6.15	3.37, 5.17	4.00, 7.43	2.85, 8.08	
Length of stay in the unit					
< 1 week	34 (77.2)	11 (73.4)	11 (78.6)	12 (80.0)	0.936 ^b^
1–2 weeks	5 (11.4)	2 (13.3)	1 (7.1)	2 (13.3)	
> 2 weeks	5 (11.4)	2 (13.3)	2 (14.3)	1 (6.7)	
ECOG performance scale					
1	4 (9.1)	1 (6.7)	1 (7.1)	2 (13.3)	0.955 ^b^
2	7 (15.9)	2 (13.3)	3 (21.4)	2 (13.3)	
3	28 (63.6)	10 (66.7)	8 (57.1)	10 (66.7)	
4	5 (11.4)	2 (13.3)	2 (14.3)	1 (6.7)	

*SD* Standard deviation; *CI* confidence interval; ^a^one-way ANOVA; ^b^Chi-square test; ^c^Fisher’s exact test

### Symptom assessment of groups

There was no significant difference between the groups for the MSAS total or subdimension mean scores before the intervention, and it was observed that the symptom load was homogeneously distributed (*p* > 0.05).

There was no significant difference between the mean MSAS total and subscale scores of the control group (*p* > 0.05). Similarly, no significant difference was found between the mean scores of MSAS-Physical and MSAS total scores within and between the groups (*p* > 0.05).

When the mean MSAS-GDI subscale scores were analyzed, a significant decrease of 7.60% was found between the mean pretest and posttest scores of the intervention group (*p* = 0.007). There was a significant decrease of 2.68% between the pre-test and post-test mean scores of the placebo group (*p* = 0.017).

When the mean MSAS-Psycho subscale scores were analyzed, there was a statistically significant decrease of 22.88% between the mean pretest and posttest scores of the intervention group (*p* = 0.003). There was a significant decrease of 13.95% between the pre-test and post-test mean scores of the placebo group (*p* = 0.007) (Table 2).

**Table 2:** Symptom assessment of groups

	Groups	Intervention (*n* = 15) mean, SD	Placebo (*n* = 14) mean, SD	Control (*n* = 15) mean, SD	*Χ*^2^	*p* value
MSAS-GDI	Pre-test	1.58, 0.56	1.49, 0.32	1.52, 0.29	0.218	0.897
	Post-test	1.46, 0.50	1.45, 0.27	1.50, 0.28	0.461	0.794
	*Z*	− 2.718	− 2.379	− 1.841		
	*p*	0.007*	0.017*	0.066		
	*Δ*	− 7.60%	− 2.68%	− 1.31%		
MSAS-Physical	Pre-test	1.40, 0.65	1.41, 0.68	1.41**,** 0.63	0.097	0.953
	Post-test	1.43, 0.62	1.40**,** 0.67	1.41**,** 0.52	0.356	0.837
	*Z*	− 1.099	− 0.730	− 0.942		
	*p*	0.272	0.465	0.346		
	*Δ*	+ 2.14%	− 0.71%	0%		
MSAS-Psycho	Pre-test	1.18, 0.70	1.29, 0.62	1.28, 0.59	0.556	0.757
	Post-test	0.91, 0.46	1.11, 0.48	1.32, 0.58	5.369	0.068
	Z	− 2.977	− 2.695	− 1.705		
	p	0.003*	0.007*	0.088		
	*Δ*	− 22.88%	− 13.95%	+ 3.13%		
MSAS-Total	Pre-test	1.07, 0.48	1.13, 0.47	1.10, 0.44	0.715	0.699
	Post-test	1.06, 0.45	1.12, 0.47	1.11, 0.43	0.246	0.884
	*Z*	− 0.881	− 1.468	− 0.395		
	*p*	0.378	0.142	0.693		
	*Δ*	− 0.93%	− 0.88%	+ 0.91%		

*MSAS* Memorial Symptom Assessment Scale; *SD* standard deviation; *Δ* percentage of change; *Z* Wilcoxon signed-ranks test; *Χ*^2^ Kruskal–Wallis *H* test; *p < 0.05; **p < 0.001

### Life satisfaction scores of groups

When the CLAS pretest mean scores of the groups were compared, no significant difference was found between the groups, and they were homogeneous (*p* > 0.05) (Table 3).

**Table 3:** Life satisfaction scores of groups

	Contentment with Life Assessment Scale					
	Pre-test mean, SD	Post-test mean, SD	Δ	*t*	*p* value	Paired differences mean, SD, %95 CI [LL, UL]
Intervention (*n* = 15)^1^	16.27, 2.68	21.66, 2.37	+ 33.12%	− 7.155	< 0.001**	− 5.40, 2.92, [− 7.02, − 3.78]
Placebo (*n* = 14)^2^	16.14, 2.31	17.21, 2.15	+ 6.63%	− 2.206	0.046*	− 1.07, 1.82, [− 2.12, − 0.02]
Control (*n* = 15)^3^	16.20, 2.35	15.40, 1.91	− 4.93%	1.922	0.075	0.80, 1.61, [− 0.09, 1.69]
*F*	0.031	16.817				
*p*	0.990	< 0.001**				
Post hoc	––	1 > 2.3				
	Group time interaction					
	Group	Time	Group*time			
*F*_2_	6.342	32.376	31.195			
*p*	0.004*	< 0.001**	< 0.001**			
*η*^2^	0.236	0.441	0.603			
Post hoc	1 > 2,3					
	pre-test < post-test					

*SD* Standard deviation; *Δ* percentage of change; *t* paired-samples *t* test; *F* one-way ANOVA; *CI* confidence interval; *F*_*2*_ repeated measures ANOVA; *η*^*2*^ Eta-square; *p < 0.05; **p < 0.001

When the mean CLAS scores of the groups were analyzed, there was a 33.12% increase between the pre-test and post-test mean scores of the intervention group, and it was significant (*p* < 0.001). There was a significant increase of 6.63% between the pre-test and post-test scores of the placebo group (*p* = 0.046). In the control group, although not significant, it indicated a decrease of 4.93% (*p* = 0.075).

When the CLAS mean posttest scores of the groups were compared, there was a significant difference (*p* < 0.001). According to post hoc analysis, the significant difference was due to the intervention group. According to repeated measures analysis, the change in CLAS scores between groups over time was statistically significant and explained 23.6% of the change in CLAS scores (*p* = 0.004). The CLAS scores of the groups also showed a significant change over time (*p* < 0.001); this change over time was found to explain 44.1% of the difference in CLAS scores. In further statistical analysis, the interaction between group and time was also found to be statistically significant (*p* < 0.001) (Table 3).

### Self-transcendence scores of groups

The comparison of the groups’ pre-test and post-test mean scores from the STS between the groups is shown in Table 4. As can be seen, there was no significant difference between the groups’ STS pre-test mean scores and they were homogeneously distributed (*p* > 0.05).

**Table 4:** Self-transcendence scores of groups

	Pre-test mean, SD	Post-test mean, SD		Δ	*t*	*p* value	Paired differences mean, SD, %95 CI [LL, UL]
Intervention (*n* = 15)^1^	35.80, 4.36	43.93, 5.39		+ 22.71%	− 14.321	< 0.001*	− 8.13, 2.20, [− 9.35, − 6.92]
Placebo (*n* = 14)^2^	36.28, 5.10	38.50, 6.00		+ 6.12%	− 2.090	0.057	− 2.21, 3.96, [− 4.50, 0.07]
Control (*n* = 15)^3^	35.53, 4.94	35.60, 5.86		+ 0.20%	− 0.065	0.949	− 0.07, 3.99, [− 2.28, 2.14]
*F*	0.244	10.312					
*p*	0.784	< 0.001**					
Post hoc	––	1 > 2,3					
	Group time interaction						
	Group	Time	Group*time				
*F*_2_	4.177	43.870	21.616				
*p*	0.022*	< 0.001**	< 0.001**				
*η*^2^	0.169	0.517	0.513				
Post hoc	1 > 2,3 pre-test < post-test						

*SD* Standard deviation; *Δ* percentage of change; *t* paired-samples *t* test; *F* one-way ANOVA; *CI* confidence interval; *F*_2_ repeated measures ANOVA; *η*^*2*^ Eta-square; *p < 0.05; **p < 0.001

When the mean STS scores of the groups were analyzed, there was a 22.71% increase between the pre-test and post-test mean scores of the intervention group, and it was significant (*p* < 0.001). There was an increase of 6.12% between the pre-test and post-test mean scores of the placebo group, but it was not statistically significant (*p* = 0.057). There was no significant difference between the pre-test and post-test mean scores of the control group (*p* = 0.949).

There was a significant difference between the STS posttest mean scores of the groups (*p* < 0.001), and this difference was due to the intervention group. According to repeated measures analysis, the change in STS scores between groups over time was statistically significant (*p* < 0.001). While groups explained 16.9% of the change (*p* = 0.022), 51.7% was explained by time (*p* < 0.001) (Table 4).

## Discussion

To our knowledge, this is the first study in a palliative care unit to comprehensively examine the effect of individual RT on the stated concepts, in which multiple sessions were applied over a short time. The findings of this randomized placebo-controlled trial showed that when compared to UCI and standard care, RT effectively reduced the mental and general distress symptoms of palliative care patients and significantly increased their level of life satisfaction and self-transcendence. This shows that the positive psychological and neurological effects of RT on well-being and emotion may also be effective in palliative care patients. Therefore, the results obtained from our study support the development of RT in an individual or standardized manner and the implementation of RT in the treatment protocols of palliative care patients.

We investigated the effects of RT by structuring it individually based on the information we received from the patient. In addition, we conducted an UCI that did not include memories, although we had patient–nurse communication and current issues to distinguish the reminiscence of positive memories from the effect of patient–nurse interaction. There was reasonably high agreement across all groups (91.7%), and no patients voluntarily withdrew from the study. According to the feedback we received from the patients, the reminiscence and social interview sessions were acceptable. The reasons some palliative care patients had to withdraw (8.3%) had to do with health conditions that could occur with the worsening of their prognosis.

Although the field of palliative care was expanded in the early 1990s when it was determined that patients in the advanced stages of many chronic fatal diseases also benefited from palliative care, most of the patients in mixed services, as in our study, were cancer patients. These patients experience many symptoms, and managing the symptom burden can be very difficult for these patients. For this reason, patients’ daily life activities are limited and indirectly their performance levels and quality of life decrease [23]. We did not find a similar study in the literature, but Ando et al. reported in their study that a short-term individual life review applied to terminal cancer patients resulted in a significant increase in life satisfaction and well-being levels compared to the control group [16]. Kwan et al. found no significant difference between the intervention and control groups in the life review used in palliative care patients, although there was an improvement in the anxiety and depression levels of the intervention group [9]. However, as in our study, interacting with patients receiving treatment in the clinic every other day, with the help of a facilitative nurse, may reduce general distress and mental symptoms. However, there was a more significant decrease in the intervention group than in the placebo group. In this context, it can be said that directing the individual’s focus to remembering the moments in the past when the patient was happy, satisfied, and proud is more effective than UCI.

Our study showed significant increases in life satisfaction levels in the intervention and placebo groups. However, although it is not very large, it may be important not to overlook the decrease in the control group (4.9%). We examined the group-time interaction, and groups explained 23.6% of the variation over time. The group*time interaction was 60.3%. In a study, it was reported that the group effect was approximately 70% in the reminiscence intervention applied to older people with dementia [5]. In addition, eight sessions of social interviews and interaction with the patients within the scope of nursing care may explain the increase in the life satisfaction level of the placebo group. Haugan et al. reported that nurse-patient interaction was significantly associated with self-transcendence and meaning of life [24]. However, the fact that the intervention group had a higher level of life satisfaction than the social interview group may be the result of a more practical intervention of RT to increase life satisfaction.

The mean STS scores obtained from the palliative care patients participating in our study were below the mean (37.5) in all three groups. STS scores were higher than our results in studies conducted on younger patients with outpatient cancer [7, 8].

In our study, STS scores increased by 22.70% in the intervention group and 6.12% in the placebo group. However, there was a significant difference only in the intervention group, and the group–time interaction was 51.3%. A study involving outpatient chemotherapy patients with a mean age of less than 60 years reported that a mind map–based life review program applied to patients increased the mean STS score from 44.72 to 52.28 (~ 16%) [8]. Although this rate in our study is higher, the difference between posttest scores may be because our sample consists of end-of-life patients. We could not find any study in the literature examining the effect of RT on the self-transcendence level of palliative care patients. Sun et al. reported in their meta-analysis that RT significantly affected cancer-related symptoms such as anxiety and depression. They also argued that RT for cancer survivors can effectively improve quality of life, self-hope, and self-esteem [6]. On the other hand, Zheng et al. applied a WeChat-based life review program to digestive tract cancer patients, and as a result, significant increases in hope and self-transcendence levels were reported in all follow-up sessions compared to the placebo (friendly visit) and control groups [25]. Zhang et al. reported that brief reminiscence-based psychosocial interventions can significantly reduce anxiety and depressive symptoms and increase hope and spiritual well-being in cancer patients, according to a meta-analysis of 20 studies involving 1744 cancer patients [26]. In addition, Babaei et al. stated that virtual RT applied to gastric cancer patients undergoing chemotherapy can reduce anxiety and depression and that RT can be a supportive psychological method for these patients [27]. Considering that the majority of our study’s sample consisted of cancer patients, as in other studies, our results were compatible with the literature.

In this context, it can be said that the fact that RT caused a significant increase in the level of self-transcendence and that there was no significant change in other groups demonstrates the effectiveness of RT for palliative care patients as well. From these results, the concept of self-transcendence can inspire healthcare professionals to take a new approach to promote and support the maintenance of health, well-being, and independence of older people and people with chronic illnesses. However, studies with larger samples in different societies are needed to fully understand the effectiveness of RT in palliative care patients.

### Strengths and limitations

The strengths of this study include there is no similar study in the literature involving patients receiving palliative care, the sessions are conducted individually, and there is an active comparator (unstructured social interviewing) group. In addition, the eight sessions were completed in 2 weeks (in the patient’s room in the hospital and did not require additional effort, time, and cost to participate in the study), the results were obtained using proven tools (MSAS, CLAS, STS), there were no side effects, and the intervention was implemented by a specialist nurse are the strengths of the study. Although the risk of deterioration in the general condition of palliative care patients, referral to intensive care, or death is high, the main strengths of our study are the low rate of data loss in this study and the fact that no patient wanted to leave the study voluntarily.

One limitation of this study was that the data were collected by a single researcher at a single center; thus, the study did not make it possible to blind them to the interventions. Another limitation is that the study was conducted with a limited number of patients admitted to the clinic. The decrease in patient circulation slowed down the data collection process as it coincided with the COVID-19 pandemic period. Another limitation is that participants in the placebo and control groups did not receive any valid intervention after the study.

## Conclusions

Our study showed that the reminiscence therapy performed in multiple sessions over a short period effectively controlled the severity of the symptoms causing mental and general distress, life satisfaction, and self-transcendence in palliative care patients. As a complementary and integrative health intervention, reminiscence therapy can be implemented by nurses and other health professionals in multiple treatment protocols to improve the well-being of palliative care patients. However, studies with larger samples are needed to fully understand the effectiveness of reminiscence therapy in palliative care patients.
